# Body composition of older adults with normal body mass index. Cross-sectional analysis of the Toulouse Frailty clinic

**DOI:** 10.1016/j.tjfa.2024.100003

**Published:** 2025-01-01

**Authors:** J. Chapelon, S. Sourdet, D. Angioni, Z. Steinmeyer, M. Briand, Y. Rolland, G. Abellan van Kan

**Affiliations:** IHU HealthAge, Frailty Clinic, Toulouse University Hospital, Gérontopôle, La Cité de la Santé, Hôpital La Grave, Place Lange, Toulouse 31059, France

**Keywords:** BMI, Dual-energy X-ray absorptiometry, Mini-nutritional assessment

## Abstract

**Background:**

Body mass index (BMI) determines general corpulence and health, whatever age, sex or clinical background. Normal BMI (18.5–24.9 kgm^2^) is defined as healthy, normal, weight leading to a false impression that no intervention is needed.

**Objectives:**

Assess the prevalence of body impairments in the presence of normal BMI.

**Design:**

Cross-sectional design. Bivariate and a multivariate regression analysis assessed the association of body composition with clinical parameters in the presence of normal BMI.

**Setting:**

Community dwelling older adults attending the Toulouse Frailty Clinic at the University Hospital, Toulouse.

**Participants:**

876 community dwelling, autonomous older adults, 70 years and over.

**Measurements:**

Dual X-ray Absorptiometry (DXA) assessment, and cognitive, physical, nutritional, and demographic evaluations were included in the present analysis.

**Results:**

Of the initial sample, 347 (39.61 %) patients had normal BMI, and among them, 152 (43.80 %) had low lean mass, 144 (41.49 %) were osteoporotic and 2 (0.58 %) increased fat mass. A poor nutritional status (Mini-Nutritional Assessment score, MNA-score, <24) was the only independent variable associated with body impairments in the presence of normal BMI (Odd Ratio 2.83; 95 % Confidence Interval 1.64–4.89).

**Conclusion:**

Nearly 70 % of the adults with normal BMI had at least one impairment in body composition (low lean mass, osteoporosis, or obesity). In the light of the present study, older adults with normal BMI and an MNA-score under 24 should be assessed with DXA to identify the age-associated impairments in body composition in order to lead to specific interventions.

## 1 Introduction

Body mass index (BMI) is an easy-to-use, internationally validated tool, implemented and recognized by the World Health Organization (WHO) since 1995, used to evaluate global corpulence and screen for weight categories that may lead to health problems using established health-related cut-points. BMI is largely used by online calculators, clinicians and researchers to characterize adults based on their weight and height and has shown to be associated with increased risk of dependency and mortality in older adults [1]. However, older adults can be misclassified since BMI does not reflect body composition, and so intervention to prevent the onset of dependency is delayed in the presence of normal BMI, in the contrary, an obesity paradox can be observed where lower mortality rates are observed in the presence high BMI [1]. Moreover, de Lorenzo [2], has shown that BMI was not a sufficient indicator to detect sarcopenia in people with an excess of fat. This field has recently been focused with the awareness on the fact that the misclassification using BMI has health implications. Indeed, patients with normal BMI and high percentage body fat, termed normal weight obesity, NWO, had higher risk of impaired physical performance, cardiometabolic dysregulation, endothelial dysfunction, insulin resistance, dyslipidaemia metabolic syndrome, and mortality [3]. Although there is still a lack of consensus on diagnostic criteria, a prevalence of 9–36 % for NWO was found, prevalence that seems to increase with age [[4], [5], [6]].

Assessing body composition (lean, fat and bone mass) is essential for the case-finding of sarcopenia, osteoporosis, and obesity. Nowadays, several techniques for the assessment of body composition exist, but it is generally agreed that Dual-energy X-ray absorptiometry (DXA) is the” gold-standard” when applied in everyday clinical practice and in research [7,8]. Using a very low dose of radiation, DXA gives information about bone mineral density (BMD), lean mass (LM), and fat mass (FM), giving total body percentages or even percentages per region of interest.

Sarcopenia, osteoporosis and obesity are age-related conditions defined by body composition changes in muscle mass and quality, bone mass and fat mass, that lead to serious adverse events. These conditions are usually associated with low BMI (<18.5) or elevated BMI (>25). But normal BMI (18.5–24.9) could give a false impression of healthy, normal, weight and so underestimate the prevalence of body composition impairments, especially in older adults. The normal range of BMI is also an ethnicity-related parameter and ranges might be different in some other ethnicities.

The age-related wasting of muscle mass, termed sarcopenia is universally present, and can be prevented or slowed down by early diagnosis and intervention. The adverse events of presenting a low muscle mass are multiple, including falls, onset of dependency, impaired quality of life, and increased mortality [[9], [10], [11]]. As shown in a recent revision, sarcopenia is associated with shortened survival, increased toxicities, and risk of recurrence in patients suffering from cancer [12]. In most statistical analysis, in order to assess if sarcopenia is associated with low or high BMI, normal BMI is used as the reference-class assuming that normal BMI is not associated with sarcopenia [13,14,15]. As for NWO, the term normal weight sarcopenia, NWS, could be used to identify patients with normal BMI and low lean mass. BMI cut points were implemented by WHO, and are used by clinicians to diagnose, to stratify the severity, and to tailor the management of obesity [16,17]. Obesity increases drastically the presence of comorbidity, hypertension, diabetes, or even respiratory diseases [18]. Even if BMI is used as indicator of overall body fat, it is not a good indicator of fat mass, lacking accuracy and reliability [16]. The presence of sarcopenia when facing obesity (sarcopenic obesity) has already been addressed in literature as a main risk factor for dependency and mortality [19]. Recent data also suggest that sarcopenia is highly prevalent in NWO-patients the same as low bone mineral density, BMD [2,20]. The presence of osteoporosis is detected through DXA measurements of BMD. Older adults are confronted with an increasing number of falls, and fragility-fractures may occur more often in the presence of osteoporosis. Age-related impairments of muscle function presenting less mass, strength, and power [21], lead to an even more increased fall-risk. In literature, BMD as been associated with low or high BMI, when once more, normal BMI was used as the reference class [22,23].

Although BMI is a relevant clinical parameter, it's known to be insufficient to identify older adults at risk of losing their functional autonomy. Moreover, normal BMI could lead to a false knowledge of a good health status with an optimal body composition. Many times, interventions are proposed for low and high BMI, with no specific considerations for normal BMI [24,25].

Therefore, the aim of this article is to assess the prevalence of impairments in body composition in a population of older adults considered as having a healthy, normal weight (normal BMI) and so, identify older adults in the need of specific interventions. The article aims also to identify independent clinical parameters that might identify an altered body composition in the presence of normal BMI. The ultimate goal is to create awareness about the fact that BMI could underestimate the presence of body composition impairments in older adults.

## 2 Material and method

### 2.1 Participants

The data used in this study were from patients from the Toulouse Frailty Clinic. A total of 876 patients were included, aged 70 years and older, with an assessment of body composition parameters by the means of a Dual X-ray absorptiometry assessment, GE-Healthcare iDXA [26]. This database has already been used to assess many clinical aspects of older adults (including lean mass and Mini Nutritional Assessment, MNA), and the methodology has already been published [27,28]. All iDXA were performed from June 5th, 2013 to January 28th, 2020.

### 2.2 Assessment of lean mass by iDXA

Muscle impairment was assessed using the operational definition proposed by Baumgartner, i.e., appendicular lean mass (ALM), sum of the lean mass (fat-free and bone-free mass) of arms and legs in kilograms, standardized by squared height in meters, ALM/HT^2^ [9]. The cut-points for low lean mass (5.5 kg/m 2 for women and 7 kg/m2 for men) were based on the European Working Group on Sarcopenia in Older People revised consensus (EWGSOP2) [11].

### 2.3 Assessment of fat mass by iDXA

Total body fat mass was used to define obesity. The percentage of body fat references for iDXA were described by Imboden [29]. Based on the article, women over 70 years old should not exceed 48.2 % of total fat mass while men should not exceed 37.4 % total fat mass. Both percentages identify the percentile-80 in a Caucasian older adult population of reference.

### 2.4 Assessment of bone mineral density by iDXA

The T-score was assessed by BMD in 3 areas (vertebrae and bilateral femoral neck). A patient was considered osteoporotic if the T-score between vertebrae L1-L4, right or left femoral neck was -2.5 or lower based on the WHO Study Group [30].

### 2.5 Assessment of BMI

For the study, the value of weight and height were clinically determined with a scale of approximately 0.1 kg and with a wall-mounted stadiometer precision1 mm respectively. Subjects were just wearing light clothes, and pieces of jewellery or watches were removed. The patients were not necessarily fasting when measurements were taken. The 876 patients were classified into 4 groups according to their BMI [1,2]: underweight, (BMI < 18.5 kgm^2^), normal (18.5–24.9 kgm^2^), overweight (25–29.9 kgm^2^), and obese (30 and over).

### 2.6 Other parameters

Clinical data were also collected from participants. Cognition was assessed by the means of the Mini Mental State Examination (MMSE) [31]. Nutritional assessment was performed using the MNA, a scale composed of 18 items including anthropometric measurements, clinical parameters, a brief dietary assessment and a self-perception about health and nutrition. For the purpose of this study, the population was dichotomized into patients presenting a poor nutritional status or at-risk (MNA score <24 points), with a good nutritional status (MNA score ≥24) [32]. Autonomy and functional capacity were estimated with the Short Physical Performance Battery (SPPB) [33] and the activities of Daily Living scale (ADL) [34]. Handgrip strength was assessed using the handheld Jamar dynamometer (Jamar, Irvington, NY, 215 USA). Each subject performed the test twice with each hand in a sitting position and the best result obtained was included [35]. Comorbidity burden was assessed using the Charlson Comorbidity index (CCI), not adjusted by age [36].

### 2.7 Statistical Analysis

All statistical analyses were performed using STATA v11 (Stata Corp., College Station, TX). Continuous variables were assessed with mean and standard deviation and the categorical ones with frequencies and percentages. Based on BMI categories, we performed a bivariate analysis with appropriates tests based on normality of distributions, *p*-value were significant below 0.05. Participants with a normal BMI were divided into two groups: those with at least one body composition impairment and those without any impairment. A stepwise backward multivariate logistic regression analysis assessed the independent variables, determining the Odd Ratio (OR) and the 95 % Confidence Interval (95 %CI). The multivariate models included all bivariate variables with a *p*-value of 0.3.

## 3 Results

Of a total of 938 patients with a DXA assessment attending the frailty clinic (62 were excluded because of their age under 70 years), 876 (93.39 % of the sample) were included with a mean age of 83.13 ± 5.91 years, 566 (64.6 %) were women.

Table 1 summarizes the participants’ characteristics classified according to their BMI. In the sample, 34 (3.88 %) of the total population was underweighted, 347 (39.61 %) had a normal BMI, 307 (35.05 %) were over-weighted and 188 (21.46 %) were obese. Sarcopenia (defined by cut-points based on ALM/HT^2^) was found in 209 patients (23.86 %), 293 (35.82 %) were osteoporotic, and 109 (12.44 %) were obese based on their percentage of body fat. Based on BMI, a good nutritional status (MNA-score of 24 or more), was more prevalent in overweight participants than in the normal BMI participants with 231 (75.24 %) and 166 (47.98 %) participants respectively.

**Table 1:** Study population.

BMI (n, %)		TOTAL 876 (100)	UNDERWEIGHT 34 (3.88)	NORMAL 347 (39.61)	OVERWEIGHT 307 (35.05)	OBESE 188 (21.46)
Gender (n, %)	Female	566 (64.61)	27 (79.41)	240 (69.16)	177 (57.65)	122 (64.89)
	Male	310 (35.39)	7 (20.59)	107 (30.84)	130 (42.35)	66 (35.11)
Age, (mean ± SD)		83.13 ± 5.91	82.95 ± 6.55	83.85 + 5.71	83.36 + 5.74	81.46 + 6.14
Weight, (mean + SD)		66.56 + 15.94	43.02 + 5.80	55.83 + 8.4	68.80 + 9.01	86.91 + 13.61
Living alone, (n, %)		407 (47.38)	23 (67.65)	158 (46.20)	132 (44.00)	94 (51.37)
College, (n, %)		520 (63.18)	23 (69.7)	210 (64.62)	179 (62.37)	108 (60.67)
Good nutrition status by MNA, (n, %)		542 (62.01)	0 (0)	166 (47.98)	231 (75.24)	145 (77.54)
MMSE ≥ 24, (n, %)		536 (63.28)	15 (45.45)	199 (58.70)	195 (66.10)	127 (70.56)
Walking speed m/s (mean + SD)		0.78 + 0.24	0.75 + 0.22	0.82 + 0.24	0.78 + 0.23	0.72 + 0.23
Hand grip strength, Kg (mean + SD)	Female	15.47 + 5.23	14.89 + 5.42	14.79 + 4.97	15.54 + 5.20	16.84 + 5.49
	Male	26.84 + 7.93	21.17 + 4.59	25.59 + 7.82	27.02 + 8.14	29.08 + 7.39
SPPB (0/12), (n, %)	Good 10-12	312 (35.7)	10 (29.41)	138 (39.88)	106 (34.53)	58 (31.02)
	Average 7-9	281 (32.15)	11 (32.35)	114 (32.95)	100 (32.57)	56 (29.95)
	Bad 0-6	281 (32.15)	13 (38.24)	94 (27.17)	101 (32.9)	73 (39.04)
ADL (mean + SD)		5.52 + 0.69	5.52 + 0.64	5.57 + 0.59	5.56 + 0.7	5.37 + 0.82
Charlson index (mean + SD)		1.27 + 1.57	0.94 + 1.13	1.08 + 1.38	1.45 + 1.75	1.41 + 1.60
Sarcopenia (n, %)		209 (23.86)	27 (79.41)	152 (43.80)	29 (9.45)	1 (0.53)
ALM, in kg (mean + SD)		17.14 + 4.21	13.43 + 2.49	15.2 + 3.23	17.73 + 3.77	20.43 + 4.26
Osteoporosis, t-score < -2.5, (n, %)		293 (35.82)	26 (81.25)	144 (41.49)	99 (33.67)	24 (13.64)
Obesity (FM percentile ≥80 (n, %)		109 (12.44)	0 (0.0)	2 (0.58)	18 (5.86)	89 (47.34)

*Legend:* BMI stands for body mass index, SD for standard deviation, MNA for mini-nutritional assessment, MMSE for mini-mental state examination, SPPB for short physical performance battery, ADL for activities of daily living, ALM for appendicular lean mass, and FM for fat mass

Participants with a normal BMI were divided into two groups: those with at least one body composition impairment (presence of low lean mass, osteoporosis, or obesity) which represents 240 patients (69.16 %, 161 were women (67.08 %)), and those without any impairment (107 patients, 30.84 %, 79 were women (73.83 %)). Into detail, 152 (43.80 %) were identified as NWS, presenting low lean mass, 144 (45.57 %) were osteoporotic, and 2 (0.58 %) were identified as NWO. Table 2 gathers the results of the bivariate analysis. We observed that the impaired group was older, had a lower walking speed, presented lower scores in SPPB and MNA. On the other hand, parameters such as gender, living arrangement or education, MMSE, handgrip strength, ADL, or the Charlson index were not statistically significant.

**Table 2:** Bivariate analysis based on the presence of impairments in body composition.

n = 347 patients		Normal BMI without impairments in body composition (n = 107, 30.84 %)	Normal BMI with at least one impairment in body composition (n = 240, 69.16 %)	p
Gender	Male (n, %)	28 (26.17)	79 (32.92)	0.209
	Female (n, %)	79 (73.83)	161 (67.08)	
Age (mean + SD)		82.57 + 5.67	84.43 + 5.64	<0.05
living alone (n, %)		43 (40.57)	115 (48.73)	0.161
College (n, %)		69 (69.70)	141 (62.39)	0.205
Good nutrition status by MNA (n, %)		72 (67.29)	94 (39.33)	<0.001
MMSE ≥ 24 (n, %)		68 (64.76)	131 (55.98)	0.129
Walking speed m/s (mean + SD)		0.87 + 0.26	0.79 + 0.23	<0.05
Hand grip strength kg (mean + SD)		18.97 + 8.02	17.76 + 7.69	0.18
SPPB	Good 10-12	54 (50.47)	84 (35.15)	<0.05
	Average 7-9	32 (29.91)	82 (34.31)	
	Bad 0-6	21 (19.63)	73 (30.54)	
ADL ≥ 5		98 (92.45)	215 (89.96)	0.461
Charlson index (mean + SD)		1.17 + 1.30	1.04 + 1.42	0.42

*Legend:* BMI stands for body mass index, *SD* for standard deviation, *MNA* for mini-nutritional assessment, *MMSE* for mini-mental state examination, *SPPB* for short physical performance battery, *ADL* for activities of daily living

Table 3 reported the results from the logistic multivariate regression analysis. After a stepwise backward multiple logistic regression analysis, MNA <24 was the strongest independent variable associated with the presence of a body composition impairment in participants with normal BMI (OR 2.83, 95 %CI [1.64–4.89]). Older age, was statistically significant in the final model with and OR of 1.05 and a 95 % CI of 1.01–1.09.

**Table 3:** Logistic regression of normal BMI with body composition impairment.

	Full model Odd ratio, 95 % IC	*p*-value	Stepwise backward model Odd Ratio, 95 % CI	*p*-value
Age	1.04 [0.99–1.09]	0.14	1.05 [1.01–1.09]	<0.05
Living alone	0.79 [0.47–1.34]	0.38	–	
College	0.96 [0.54–1.71]	0.883	–	
MNA (score < 24)	2.82 [1.63–4.87]	<0.001	2.90 [1.74–4.81]	<0.001
MMSE (score ≥ 24)	1.16 [0.65–2.04]	0.62	–	
Walking speed	0.59 [0.14–2.07]	0.48	–	
Handgrip strength	1.00 [0.97–1.04]	0.79	–	
SPPB	1.08 [0.56–2.07]	0.82	–	
ADL ≥ 5	1.33 [0.52–3.40]	0.56	–	

*Legend:* BMI stands for body mass index, *CI* for Confidence Interval, *MNA* for mini-nutritional assessment, *MMSE* for mini-mental state examination, *SPPB* for short physical performance battery, *ADL* for activities of daily living.

## 4 Discussion

The present analysis show that the use of BMI was not sufficient to conclude on the general state of health of older adults. We show that in the presence of normal BMI, nearly 70 % of the participants present at least one impairment in body composition, with 43.80 % suffering from low lean mass, and 41.49 % from osteoporosis. Multivariate analysis showed that age (OR: 1.05; 95 %CI 1.01–1.09) and MNA under 24 (OR: 2.90; 95 %CI 1.74–4.81) were the associated factors with presence of impaired body composition in the normal BMI subgroup.

Several studies have shown that BMI alone is not a sufficient indicator of healthy body composition. In obese patients, Frankenfield et al. showed that the use of BMI underestimated obesity when compared to the presence of total fat mass measured by bioelectrical impedance [37]. The study concluded that BMI could misclassify obese patients when BMI is below 30 kgm^2^. Cross-sectional analysis performed by Woo et al, showed that both high fat mass and low appendicular lean mass (assessed by DXA) were significantly associated with dependency and low grip strength independently of BMI groups [38]. These studies, in the same line as ours, show that BMI does not reflect accurately total body fat. The fact to use percentile-80 (>48.2 % of total fat mass for women and >37.4 % for men) to establish obesity has dampened the association between obesity and normal BMI [29] in our study. NWO-participants in the NHANES III study were identified as having body fat of >40.3 % for women and 28 % for men; a much lower threshold [3]. The prevalence of NWO is about 9–36 % among older adults, increasing with age and presenting a higher risk of associated sarcopenia [[4], [5], [6]]. We could hypothesise that the established cut-points of BMI are not valid for an elderly population. Indeed, different academic societies in the field of aging and nutrition, like the European Society of Clinical Nutrition (ESPEN) or the GLIM criteria [39], have proposed alternative threshold for BMI in older adults (low BMI is <20 or <22 kgm^–2^ in subjects younger and older than 70 years, respectively). Nevertheless, since these published papers in 2015, the WHO BMI-criteria have not been adapted, current online calculators do not consider age when calculating BMI, and health carers and researchers keep using the classical thresholds to manage nutritional issues. Even if being aware, it was a methodological choice in the present paper to use the normal BMI range as proposed by WHO (18.5–24.9 kgm^–2^) in order to create awareness of an erroneous perception of healthy composition in older adults.

Malnutrition is associated with low BMI (low weight) and sarcopenia [40]. This is the first time that data are shown on normal weight patients presenting sarcopenia showing that NWS should become an active domain of inquiry, in the same line as NWO. Moreover, as shown by Burman et al., BMI underestimates the presence of malnutrition [41]. Although malnutrition and BMI were associated in their study, they concluded on a misclassification using BMI, and many patients with a low MNA score still had a high BMI value, and vice versa. Misclassification (using BMI) of malnourished patients identified by MNA is an important issue as malnutrition has been associated with several geriatric syndromes (like dementia, comorbidity, incontinence, or functional decline), dependency, sarcopenia, and mortality [32,40,42,43,[44], [45], [46]].

The present analysis focusses on patients with a normal BMI, and as seen in previous studies this subgroup cannot be considered as having a healthy normal weight, indeed nearly 70 % of them presented an impairment in body composition (low lean mass, osteoporosis, or obesity). This led us to search for independent variables that could be associated with these prevalent impairments in the elderly in the presence of normal BMI. Other than age, using a stepwise backward multivariate logistic regression analysis, we found that MNA under 24 (i.e., patients without a good nutritional status) was the only associated variable. In other words, for older adults (over 70 years) with a normal BMI, before arguing the presence of a healthy weight, it could be interesting to assess their nutritional status using the MNA, and assess their body composition searching for impairments when MNA is under 24. MNA, as an initial case-finding evaluation, is less expensive and more convenient than directly assessing body composition by DXA, less available in clinical practice. The present study highlights, once more, the importance of assessing MNA in older adults. MNA and its short form have already been linked with clinical malnutrition, frailty, osteoporosis and sarcopenia, dependency and mortality [47], [48].

The main limitation of our study is its cross-sectional design. No causal relationship can be established to enlighten the association found in our study. Longitudinal studies should clarify the different hypothesis, mainly the idea of misclassification using BMI or if MNA could identify, at an early stage, the decline of intrinsic capacity. The study participants were recruited from the frailty clinic, so that our analysis need to be confirmed in general population in order to assess representativeness. Our participants were autonomous, community dwelling, older adults. The results need to be confirmed in different subgroup of more dependent or institutionalised older adult in order to be generalised, and conclude on a misclassification using BMI in older adults.

## 5 Conclusion

As a conclusion, our study enlightens the fact that BMI could misclassify older adults. Clinicians should be aware that in the presence of normal BMI, diagnosis like osteoporosis, sarcopenia, or obesity could still be present. We propose an algorithm of intervention in order to cope with this issue. Indeed, the idea to perform an MNA when normal BMI is found, followed by a detailed assessment of body composition if MNA is impaired, could lead to specific interventions to prevent dependency. In the light of the present article, we stress that clinicians should be aware of the fact that a normal BMI is not sufficient to state that patients present a healthy body weight. NWS and NWO should become of areas of interest, in order to tailor intervention when participants present normal weight identified by BMI.

## Declaration of competing interest

The authors declare that they have no known competing financial interests or personal relationships that could have appeared to influence the work reported in this paper.

## References

[1] Dramé M, Godaert L (2023). The obesity paradox and mortality in older adults: a systematic review. Nutrients.

[2] De Lorenzo A, Pellegrini M, Gualtieri P, Itani L, El Ghoch M, Di Renzo L (2022). The risk of sarcopenia among adults with normal-weight obesity in a nutritional management setting. Nutrients.

[3] Batsis JA, Sahakyan KR, Rodriguez-Escudero JP, Bartels SJ, Lopez-Jimenez F (2014). Normal weight obesity and functional outcomes in older adults. Eur J Intern Med.

[4] Ji T, Zhang L, Tang Z, Sun F, Li Y, Ma L (2020). Prevalence of normal-weight obesity in community-dwelling chinese older adults: results from the Beijing longitudinal study of aging. Diabetes Metab Syndr Obes.

[5] Franco LP, Morais CC, Cominetti C (2016). Normal-weight obesity syndrome: diagnosis, prevalence, and clinical implications. Nutr Rev.

[6] Oliveros E, Somers VK, Sochor O, Goel K, Lopez-Jimenez F (2014). The concept of normal weight obesity. Prog Cardiovasc Dis.

[7] Holmes CJ, Racette SB (2021). The utility of body composition assessment in nutrition and clinical practice: an overview of current methodology. Nutrients.

[8] Rothney MP, Martin FP, Xia Y, Beaumont M, Davis C, Ergun D, Rezzi S (2012). Precision of GE Lunar iDXA for the measurement of total and regional body composition in nonobese adults. Journal of Clin Densitom.

[9] Baumgartner RN, Koehler KM, Gallagher D, Romero L, Heymsfield SB, Ross RR, Lindeman RD (1998). Epidemiology of sarcopenia among the elderly in New Mexico. Am J Epidemiol.

[10] Cooper C, Dere W, Evans W, Kanis JA, Rizzoli R, Sayer AA, Sieber CC, Kaufman JM, Abellan van Kan G, Boonen S, Adachi J, Mitlak B, Tsouderos T, Rolland Y, Reginster JYL (2012). Frailty and sarcopenia: definitions and outcome parameters. Osteoporos Int.

[11] Cruz-Jentoft AJ, Bahat G, Bauer J (2019). Writing group for the European working group on sarcopenia in older people 2 (EWGSOP2), and the extended group for EWGSOP2. Sarcopenia: revised European consensus on definition and diagnosis. Age Ageing.

[12] Zhang FM, Wu HF, Shi HP, Yu Z, Zhuang CL (2023). Sarcopenia and malignancies: epidemiology, clinical classification and implications. Ageing Res Rev.

[13] Curtis M, Swan L, Fox R, Warters A, O'Sullivan M (2023 Mar 21). Associations between body mass index and probable sarcopenia in community-dwelling older adults. Nutrients.

[14] Du Y, Tao S, Oh C, No J (2023). The function of body mass index in the older with osteosarcopenia: a systematic review and meta-analysis. J Obes Metab Syndr.

[15] Pereira CC, Pagotto V, de Oliveira C, Silveira EA (2022). Sarcopenia and mortality risk in community-dwelling Brazilian older adults. Sci Rep.

[16] Sweatt K, Garvey WT, Martins C (2024). Strengths and limitations of BMI in the diagnosis of obesity: what is the path forward?. Curr Obes Rep.

[17] (2000). Obesity: preventing and managing the global epidemic. Report of a WHO consultation. World Health Organ Tech Rep Ser.

[18] Amarya S, Singh K, Sabharwal M (2014). Health consequences of obesity in the elderly. J Clin Gerontol Geriatr.

[19] Batsis JA, Villareal DT (2018). Sarcopenic obesity in older adults: aetiology, epidemiology and treatment strategies. Nat Rev Endocrinol.

[20] Kim J, Kang S, Kang H (2024). Association between normal-weight obesity and bone mineral density in older Korean adults: a population-based cross-sectional study. Maturitas.

[21] Metter E J, Conwit R, Tobin J, Fozard J L (1997). Age-associated loss of power and strength in the upper extremities in women and men. J Gerontol Ser.

[22] Lloyd JT, Alley DE, Hawkes WG, Hochberg MC, Waldstein SR, Orwig DL (2014). Body mass index is positively associated with bone mineral density in US older adults. Arch Osteoporos.

[23] Chen C, Jia J, Wang P (2024). The saturation effect of body mass index on total lumbar bone mineral density for adults: the NHANES 2011-2020. Medicine (Baltimore).

[24] Chu YR, Xu YC, Ma LL, Wang JX, Zong HX, Tong WQ, Wang XL, Zhao X, Xu SQ (2024). Skeletal muscle index together with body mass index is associated with secondary osteoporosis in patients with rheumatoid arthritis. Eur J Med Res.

[25] (2022). Kulapong Jayanama 1 2, Olga Theou 2 3, Judith Godin 2, Andrea Mayo 2, Leah Cahill 4 5, Kenneth Rockwood 6 7] Relationship of body mass index with frailty and all-cause mortality among middle-aged and older adults. BMC Med.

[26] Kelly T L, Berger N, Richardson T L (1998). DXA body composition: theory and practice. App Radiat Isotopes.

[27] Fougère B, Sourdet S, Lilamand M, Tabue-Teguod M, Teysseyre B, Dupuy C, Vellas B, Rolland Y, Nourhashemi F, Abellan van Kan G (2018). Untangling the overlap between frailty and low lean mass: data from Toulouse Frailty day hospital. Arch Gerontol Geriatr.

[28] Lilamand M, Kelaiditi E, Cesari M, Raynaud-Simon A, Ghisolfi A, Guyonnet S, Vellas B, van Kan GA, Toulouse Frailty Platform Team (2015). Validation of the mini nutritional assessment-short form in a population of frail elders without disability. analysis of the toulouse frailty platform population in 2013. J Nutr Health Aging.

[29] Imboden M T, Welch W A, Swartz A M, Montoye A H, Finch H W, Harber M P, Kaminsky L A (2017). Reference standards for body fat measures using GE dual energy x-ray absorptiometry in Caucasian adults. PloS one.

[30] Faulkner K G (2005). The tale of the T-score: review and perspective. Osteoporosis Int.

[31] Folstein M F, Folstein S E, McHugh P R (1975). Mini-mental state”: a practical method for grading the cognitive state of patients for the clinician. JPsychiatr Res.

[32] Guigoz Y, Vellas B (1999). The mini nutritional assessment (MNA) for grading the nutritional state of elderly patients: presentation of the MNA, history and validation. Nestle Nutr Workshop Ser Clin Perform Programme.

[33] Guralnik J M, Simonsick E M, Ferrucci L, Glynn R J, Berkman L F, Blazer D G, Wallace R B (1994). A short physical performance battery assessing lower extremity function: association with self-reported disability and prediction of mortality and nursing home admission. J Gerontol.

[34] Katz S, Ford A B, Moskowitz R W, Jackson B A, Jaffe M W (1963). Studies of illness in the aged: the index of ADL: a standardized measure of biological and psychosocial function. Jama.

[35] Lee SH, Gong HS (2020). Measurement and interpretation of handgrip strength for research on sarcopenia and osteoporosis. J Bone Metab.

[36] Charlson ME, Pompei P, Ales KL, MacKenzie CR (1987). A new method of classifying prognostic comorbidity in longitudinal studies: development and validation. J Chronic Dis.

[37] Frankenfield D C, Rowe W A, Cooney R N, Smith J S, Becker D (2001). Limits of body mass index to detect obesity and predict body composition. Nutrition.

[38] Woo J, Leung J, Kwok T (2007). BMI, body composition, and physical functioning in older adults. Obesity.

[39] Cederholm T, Bosaeus I, Barazzoni R, Bauer J, Van Gossum A, Klek S, Muscaritoli M, Nyulasi I, Ockenga J, Schneider SM, de van der Schueren MA, Singer P (2015). Diagnostic criteria for malnutrition - An ESPEN consensus statement. Clin Nutr.

[40] Calcaterra L, Abellan van Kan G, Steinmeyer Z, Angioni D, Proietti M, Sourdet S (2024). Sarcopenia and poor nutritional status in older adults. Clin Nutr.

[41] Burman M, Säätelä S, Carlsson M, Olofsson B, Gustafson Y, Hörnsten C (2015). Body mass index, mini nutritional assessment, and their association with five-year mortality in very old people. J Nutr, Health & Aging.

[42] Oliveira M R, Fogaça K C, Leandro-Merhi V A (2009). Nutritional status and functional capacity of hospitalized elderly. Nutr J.

[43] Lee L C, Tsai A C H (2012). Mini-Nutritional-Assessment (MNA) without body mass index (BMI) predicts functional disability in elderly taiwanese. Arch Gerontol Geriatr.

[44] Persson M D, Brismar K E, Katzarski K S, Nordenström J, Cederholm T E (2002). Nutritional status using mini nutritional assessment and subjective global assessment predict mortality in geriatric patients. J Am Geriatr Soc.

[45] Lundin H, Sääf M, Strender L E, Mollasaraie H A, Salminen H (2012). Mini nutritional assessment and 10-year mortality in free-living elderly women: a prospective cohort study with 10-year follow-up. Eur J Clin Nutr.

[46] Saka B, Kaya O, Ozturk G B, Erten N, Karan M A (2010). Malnutrition in the elderly and its relationship with other geriatric syndromes. Clin nutr.

[47] Beaudart C, Sanchez-Rodriguez D, Locquet M, Reginster J Y, Lengelé L, Bruyère O (2019). Malnutrition as a strong predictor of the onset of sarcopenia. Nutrients.

[48] Salminen H, Sääf M, Johansson S E, Ringertz H, Strender L E (2006). Nutritional status, as determined by the Mini-Nutritional Assessment, and osteoporosis: a cross-sectional study of an elderly female population. Eur J Clin Nutr.

